# Extracellular matrix metalloproteinase inducer (EMMPRIN) expression correlates positively with active angiogenesis and negatively with basic fibroblast growth factor expression in epithelial ovarian cancer

**DOI:** 10.1007/s00432-013-1569-z

**Published:** 2013-12-28

**Authors:** Sebastian Szubert, Dariusz Szpurek, Rafal Moszynski, Michal Nowicki, Andrzej Frankowski, Stefan Sajdak, Slawomir Michalak

**Affiliations:** 1 Division of Gynecological Surgery, Poznan University of Medical Sciences, 33 Polna St., 60-535 Poznan, Poland; 2 Department of Histology and Embryology, Poznan University of Medical Sciences, Poznan, Poland; 3 Department of Pathology, Gynecologic and Obstetrical University Hospital, Poznan, Poland; 4 Department of Neurochemistry and Neuropathology, Poznan University of Medical Sciences, Poznan, Poland; 5 Neuroimmunological Unit Polish Academy of Sciences, Poznan, Poland

**Keywords:** Extracellular matrix metalloproteinase inducer, EMMPRIN, Vascular endothelial growth factor, Basic fibroblast growth factor, Ovarian cancer, Angiogenesis

## Abstract

**Purpose:**

The primary aim of this paper was to evaluate the expression of extracellular matrix metalloproteinase inducer (EMMPRIN) and its relationship with proangiogenic factors and microvessel density (MVD) in ovarian cancer.

**Methods:**

The study group included 58 epithelial ovarian cancers (EOCs), 35 benign ovarian tumors, and 21 normal ovaries. The expression of EMMPRIN, vascular endothelial growth factor (VEGF), and basic fibroblast growth factor (bFGF) was assessed by ELISA of tissue homogenates. Antibodies against CD105, CD31, and CD34 were used to immunohistochemically assess MVD.

**Results:**

We have found significantly higher EMMPRIN expression in EOC than in benign ovarian tumors and normal ovaries. Similarly, the VEGF expression was higher in EOC than in benign ovarian tumors and normal ovaries. By contrast, bFGF expression was lower in EOC than in benign ovarian tumors and ovary samples. EMMPRIN expression in EOC was directly correlated with VEGF expression and CD105-MVD, but inversely correlated with bFGF expression. Grade 2/3 ovarian cancers had increased expression of EMMPRIN and VEGF, increased CD105-MVD, and lowered expression of bFGF compared to grade 1 ovarian cancers. Moreover, EMMPRIN expression was higher in advanced (FIGO III and IV) ovarian cancer.

**Conclusions:**

The upregulation of EMMPRIN and VEGF expression is correlated with increased CD105-MVD and silenced bFGF, which suggests early and/or reactivated angiogenesis in ovarian cancer. Aggressive EOC is characterized by the following: high expression of EMMPRIN and VEGF, high CD105-MVD, and low expression of bFGF.

## Introduction

Epithelial ovarian cancer (EOC) is the leading cause of death from gynecological malignancies and the fifth leading cause of cancer-related death among women in the United States (Jemal et al. 2010). The 5-year survival rate is approximately 45.6 % (Howlader et al. 2012). Although complete remission after the primary treatment is achieved in approximately half of patients, the majority will relapse, and the disease then becomes fatal (Gadducci et al. 1998; du Bois et al. 2003). The poor prognosis of ovarian cancer patients has motivated the development of new anticancer therapies. Recently, antiangiogenic treatments have been introduced, and several trials have reported encouraging results in the management of ovarian cancer patients (Burger 2011).

Current antiangiogenic strategies are primarily based on the inhibition of vascular endothelial growth factor (VEGF). Two randomized placebo-controlled trials reported a significant response and prolonged progression-free survival (PFS) after the incorporation of bevacizumab, an anti-VEGF monoclonal antibody, into the primary chemotherapy regimen for ovarian cancer patients (Burger et al. 2011; Perren et al. 2011). However, after the discontinuation of maintenance bevacizumab therapy, the disease was exacerbated, and no improvement in overall survival was observed (Burger et al. 2011; Perren et al. 2011). In the case of recurrent or persistent ovarian cancer, bevacizumab monotherapy produced a 16–21 % response rate, and the median PFS was less than 5 months (Burger et al. 2007; Cannistra et al. 2007). Taken together, these results suggest that anti-VEGF therapy in ovarian cancer is effective but insufficient. Particularly in the primary treatment, the improvement in PFS was modest (Tomao et al. 2013; Collinson et al. 2013). The multipurpose blockade of VEGF and other proangiogenic factors may be more effective than a single anti-VEGF approach (Bergers and Hanahan 2008; Alessi et al. 2009; Burger 2011). Potential strategies include the application of multikinase inhibitors that impede the signaling of several important proangiogenic molecules, such as VEGF, platelet-derived growth factor (PDGF), and fibroblast growth factor (FGF). Such multikinase inhibitors are currently in clinical trials in ovarian cancer patients (Burger 2011).

Extracellular matrix metalloproteinase inducer (EMMPRIN), also known as basigin or cluster of differentiation 147 (CD147), is another candidate for the antiangiogenic treatment of cancer. EMMPRIN is a transmembrane protein member of the immunoglobulin family of receptors (Weidle et al. 2010). As a membrane protein, EMMPRIN has been suggested to act via cell–cell interactions with surrounding cells to stimulate the secretion of matrix metalloproteinases (MMPs) (Biswas et al. 1995). Homotypic EMMPRIN–EMMPRIN interactions are likely responsible for EMMPRIN activity (Sun and Hemler 2001; Seizer et al. 2009). However, it is not known whether direct cell–cell contact is necessary because soluble EMMPRIN and EMMPRIN-enriched tumor microvesicles also have the ability to stimulate the secretion of MMPs (Li et al. 2001; Egawa et al. 2006; Millimaggi et al. 2007).

High expression of EMMPRIN has been observed in various human neoplasms and frequently correlated with cancer aggressiveness (Zucker et al. 2001; Riethdorf et al. 2006). In cancer development, EMMPRIN stimulates the secretion of MMPs, which leads to the destruction of the extracellular matrix to facilitate cancer cell migration, metastasis, and angiogenesis (Gabison et al. 2005; Weidle et al. 2010). Furthermore, EMMPRIN also stimulates VEGF secretion (Tang et al. 2005; Voigt et al. 2009; Bougatef et al. 2009). It was also shown that EMMPRIN may enhance angiogenesis by activating the proliferation, survival, and migration of endothelial cells (ECs) (Chen et al. 2009).

Although several studies have identified EMMPRIN expression in ovarian cancer, little is known about the role of EMMPRIN in the angiogenesis of ovarian cancer. Thus, the main purpose of this study was to investigate the potential association between the expression of EMMPRIN and other proangiogenic factors with microvessel density (MVD) in ovarian cancer samples.

## Materials and methods

### Participants

The study group included 58 samples of EOC collected from women treated in the Division of Gynecological Surgery, Poznan University of Medical Sciences, Poland, during the years 2007–2012. The control group comprised 35 samples of benign ovarian tumors taken from patients who were operated on in our division. In addition, 21 samples of normal ovaries were obtained from perimenopausal women (median age 51; range 46–55 years), operated on due to non-oncological conditions. The materials obtained during surgery were divided into 2 parts. The first part was fixed in buffered formalin, and the second part was frozen just after collection and stored at −82 °C.

Tumors removed during surgery were examined histopathologically and classified according to WHO criteria. The histological types of the EOCs included in the study were as follows: 22 serous, 8 mucinous, 7 endometrioid, 4 clear cell adenocarcinomas, and 17 undifferentiated carcinomas. The group of benign ovarian tumors included the following: 10 serous and 5 mucinous cystadenomas, 7 endometrioid cysts, 10 adult teratomas, and 3 fibrothecomas. Malignant tumors were classified into three histological grades; 13, 11, and 34 tumors were classified as grades 1, 2, and 3, respectively. The clinical stage of the disease was specified using the criteria of the International Federation of Gynecology and Obstetrics (FIGO). Malignant tumors were classified according to the FIGO stage of the disease as follows: 15 stage I patients, 9 stage II patients, 24 stage III patients, and 10 stage IV patients.

This study received local ethics committee approval, and all patients signed consent forms before participating.

### EMMPRIN, VEGF, and bFGF expression

The expression of EMMPRIN, VEGF, and bFGF was assessed by ELISA of tissue homogenates. Homogenates were obtained from freshly frozen tissue samples. The analyzed tissue specimens were homogenized mechanically in a buffer containing 150 mM NaCl (Sigma Aldrich, USA), 5 mM EDTA (Sigma Aldrich, USA), 50 mM Tris–HCl, pH 7.4 (Sigma Aldrich, USA), 1 % Triton X-100 (Sigma Aldrich, USA), and a protease inhibitor cocktail (Sigma Aldrich, USA, catalogue number S8820). The homogenates were centrifuged for 15 min in Eppendorf tubes at 10,000 rpm. The supernatants were used for ELISA to measure the concentration of proangiogenic factors (VEGF, bFGF, and EMMPRIN). We used commercially available ELISA kits obtained from R&D Systems, Minneapolis, Minnesota, USA. The expression of VEGF, bFGF, and EMMPRIN is reported as the tissue protein content (pg/mg total protein). Protein concentrations were assessed according to Lowry’s method. No repeated freeze–thaw cycles were performed before ELISA analysis in any case. Before analysis, we have performed micro-dissection to exclude connective tissue and large vessels from the specimen. The samples were not batched for the analysis, and until statistical analysis, all the members of the team involved in ELISA analysis were blinded against studied groups. The analyses were performed in duplicates for each series, and following coefficients of variation (cv) were calculated for intra-assay variability: VEGF = 13 %; bFGF = 6.98 %, EMMPRIN = 11.00 %.

### Immunohistochemistry and MVD assessment

**Fig. 1:**
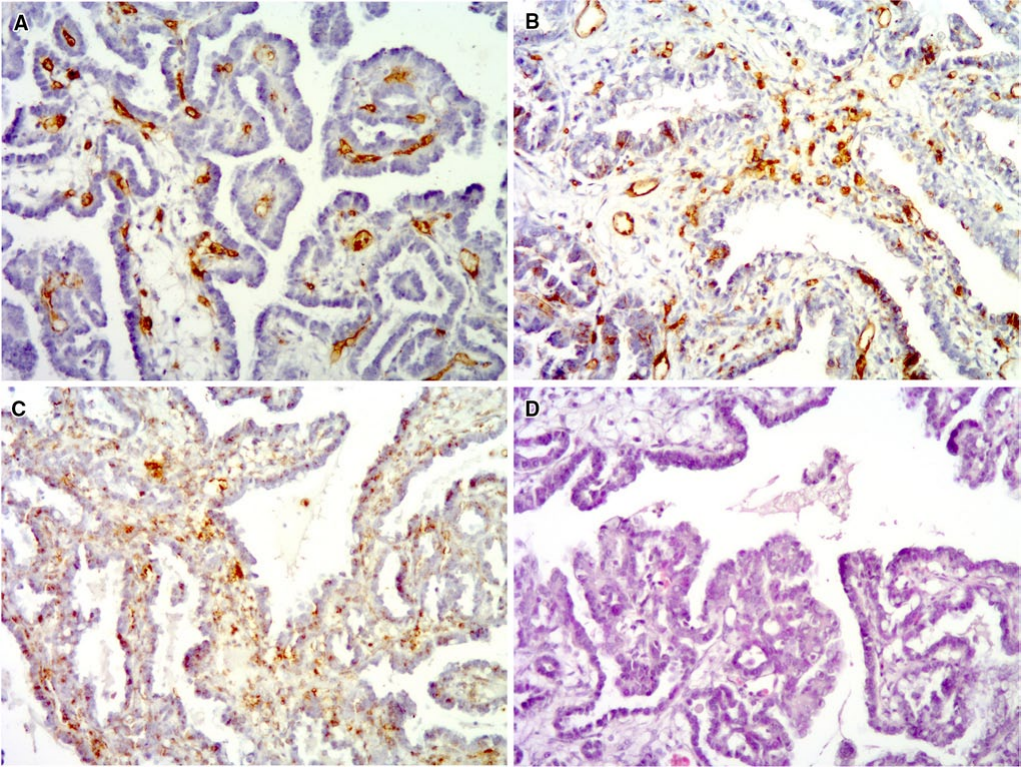
Clear cell ovarian carcinoma showing high MVD for all of the analyzed endothelial markers: CD31 (**a**), CD34 (**b**), and CD105 (**c**). Hematoxylin and eosin staining of the tumor is presented in (**d**)

Formalin-fixed tissue samples were used to evaluate MVD. Immunohistochemistry was used for endothelium labeling with antibodies against CD105, CD31, and CD34. A modified protocol proposed by Rubatt et al. (2009) was used for the assessment of MVD. Briefly, one observer screened the whole sample by light microscopy at 40× magnification to identify the three largest microvessel clusters (“hot spots”). Only hot spots located near neoplastic cells were analyzed. Subsequently, microvessels were counted in each of the selected hot spots at 400× magnification. Only microvessels with lumen were considered. Large vessels and vessels with muscular walls were not counted. The median number of microvessels from 3 hot spots was used for the final analysis. MVD assessment was conducted by histopathologist experienced in angiogenesis studies. Representative immunohistochemical staining is presented in Fig. 1.

### Statistical analysis

Statistical analysis was performed with GraphPad InStat 3.06, and following statistical tests were used: Kolmogorov–Smirnov test (K–S test), Kruskal–Wallis test (nonparametric ANOVA) with Dunn’s multiple comparisons test as a post hoc test, unpaired t test, Mann–Whitney test, and Spearman rank correlation. Logistic regression was conducted using MedCalc 11.4.2.0 software.

## Results

### VEGF, bFGF, and EMMPRIN in EOCs, benign ovarian tumors, and normal ovaries

The median EMMPRIN expression in the EOC group was significantly higher than that in the benign ovarian tumor and normal ovary groups (*P* = 0.0002). Similarly, the median VEGF expression was higher in the EOC group compared to the benign ovarian tumor and normal ovary groups (*P* < 0.0001). By contrast, the median bFGF expression was significantly lower in the EOC group compared to the benign ovarian tumor and normal ovary groups (*P* < 0.0001). These results are summarized in Table 1.

**Table 1:** Median concentration of proangiogenic factors within the three analyzed groups

	Ovarian cancer (*n* = 58)	Benign ovarian tumors (*n* = 35)	Normal ovaries (*n* = 21)	*P* value
VEGF Median pg/mg protein (range, min–max)	444.64 (0–3,000.00)	2.02 (0–634.75)	4.93 (1.67–79.35)	**<0.0001**
EMMPRIN Median ng/mg protein (range, min–max)	33.41 (8.48–106.50)	22.94 (13.26–48.38)	21.06 (10.65–40.75)	**0.0002**
bFGF Median ng/mg protein (range, min–max)	0.97 (0.17–13.32)	2.20 (0.67–50.31)	8.04 (2.56-12.91)	**<0.0001**

Bold values are statistically significant (*P* < 0.05)

### MVD in the studied groups

We observed significantly higher (*P* = 0.0003) MVD in the ovarian cancer tumors (25 vessels/mm^2^; range 0–57) than in the benign ovarian tumors (6 vessels/mm^2^; 0–70) and normal ovaries (6 vessels/mm^2^; 0–26), as assessed by CD105 staining. There were no differences in MVD as assessed by antibodies against CD31 and CD34 between the three studied groups. The results of the MVD assessment are presented in Table 2.

**Table 2:** MVD assessment in the studied group of patients and the healthy individuals

	Ovarian cancer (*n* = 58) median vessels/mm^2^ (range, min–max)	Benign ovarian tumors (*n* = 35) Median vessels/mm^2^ (range, min–max)	Normal ovaries (*n* = 21) median vessels/mm^2^ (range, min–max)	*P* value
CD105-MVD	25 (0–57)	6 (0–70)	6 (0–26)	**0.0003**
CD31-MVD	76 (19–241)	70 (6–399)	60 (31–284)	0.817
CD34-MVD	70 (6–190)	70 (6–386)	114 (31–354)	0.077

CD105-MVD, CD31-MVD, and CD34-MVD refer to MVD assessed with antibodies against CD105, CD31, and CD34, respectively

Bold value is statistically significant (*P* < 0.05)

### Proangiogenic factors expression in relation to MVD

In the EOC group, EMMPRIN expression positively correlated with MVD assessed by CD105 staining (R Spearman = 0.308, *P* = 0.031). Similarly, the expression of VEGF correlated positively with CD105-MVD (R Spearman = 0.285, *P* = 0.049). By contrast, there was no significant relationship between bFGF expression and CD105-MVD (R Spearman = −0.149, *P* = 0.314). Neither CD31-MVD nor CD34-MVD correlated with the expression of any of the analyzed proangiogenic factors. The correlation results are summarized in Table 3. In the logistic regression model, none of the three analyzed proangiogenic factors was found to be an independent prognostic factor of CD105-MVD higher than the 75th percentile (>38 microvessels/mm^2^).

**Table 3:** Correlation analyses between the expression of proangiogenic factors and MVD in the EOC group

	CD105-MVD (*P* value)	CD31-MVD (*P* value)	CD34-MVD (*P* value)
VEGF	**R Spearman = 0.285**	R Spearman = 0.005	R Spearman = −0.086
	***P*** = 0.049	*P* = 0.973	*P* = 0.577
EMMPRIN	**R Spearman = 0.308**	R Spearman = 0.193	R Spearman = 0.151
	***P*** **= 0.031**	*P* = 0.183	*P* = 0.323
bFGF	R Spearman = −0.149	R Spearman = −0.256	R Spearman = −0.167
	*P* = 0.314	*P* = 0.08	*P* = 0.278

CD105-MVD, CD31-MVD, and CD34-MVD refer to MVD assessed with antibodies against CD105, CD31, and CD34, respectively

Bold values are statistically significant (*P* < 0.05)

### Reciprocal correlations between expression of proangiogenic factors

The analysis of correlations between the expression of proangiogenic factors revealed a significant positive correlation between EMMPRIN and VEGF expression (R Spearman = 0.364, *P* = 0.006), whereas the expression of EMMPRIN was inversely proportional to bFGF expression (R Spearman = −0.28, *P* = 0.035). The correlation between the expression of bFGF and VEGF was insignificant (R Spearman = −0.248, *P* = 0.07).

### Proangiogenc factors expression and MVD regarding clinical features of EOC

The median EMMPRIN concentration was significantly higher in advanced ovarian cancer samples (FIGO III and IV) compared to early-stage tumor samples (FIGO I and II) (*P* = 0.013). Moreover, grade 2/3 ovarian cancers were characterized by the increased expression of EMMPRIN and VEGF, increased CD105-MVD, and the lowered expression of bFGF compared to grade 1 ovarian cancers (*P* = 0.026, 0.019, 0.017, and 0.018, respectively). Tables 4 and 5 show the expression of proangiogenic factors and the MVD assessment according to FIGO stage and the grade of ovarian cancer, respectively. However, we found no differences in EMMPRIN expression between various histopathological types of ovarian cancer. Similarly, there were no differences in VEGF or bFGF expression or MVD between the different histopathological types of ovarian cancer (Table 6).

**Table 4:** Expression of proangiogenic factors and MVD according to the FIGO stage of ovarian cancer

	FIGO I and II (*n* = 24)	FIGO III and IV (*n* = 34)	*P* value
VEGF Median pg/mg protein (range, min–max)	270.35 (0–2,371.3)	610.35 (10.14–3,000.0)	0.134
EMMPRIN Median ng/mg protein (range, min–max)	26.52 (8.48–90.28)	37.55 (10.65–106.50)	**0.013**
bFGF Median ng/mg protein (range, min–max)	0.88 (0.27–13.32)	1.03 (0.17–8.33)	0.966
CD105-MVD Median vessels/mm^2^ (range, min–max)	16 (0–57)	25 (0–51)	0.203
CD31-MVD Median vessels/mm^2^ (range, min–max)	63 (25–203)	76 (19–241)	0.392
CD34-MVD Median vessels/mm^2^ (range, min–max)	57 (6–164)	82 (32–190)	0.171

CD105-MVD, CD31-MVD, and CD34-MVD refer to MVD assessed with antibodies against CD105, CD31, and CD34, respectively

Bold value is statistically significant (*P* < 0.05)

**Table 5:** Expression of proangiogenic factors and MVD according to the grade of ovarian cancer

	G1 (*n* = 13)	G2/3 (*n* = 45)	*P* value
VEGF Median pg/mg protein (range, min–max)	257.76 (0–1265.8)	625.35 (10.14–3,000.0)	**0.019**
EMMPRIN Median ng/mg protein (range, min–max)	25.92 (8.48–44.25)	35.90 (10.86–106.50)	**0.026**
bFGF Median ng/mg protein (range, min–max)	2.19 (0.72–13.32)	0.82 (0.17–8.33)	**0.018**
CD105-MVD Median vessels/mm^2^ (range, min–max)	6 (0–44)	28 (0–57)	**0.017**
CD31-MVD Median vessels/mm^2^ (range, min–max)	63 (25–241)	76 (19–203)	0.223
CD34-MVD Median vessels/mm^2^ (range, min–max)	54 (6–146)	76 (32–190)	0.153

CD105-MVD, CD31-MVD, and CD34-MVD refer to MVD assessed with antibodies against CD105, CD31, and CD34, respectively

Bold values are statistically significant (*P* < 0.05)

**Table 6:** Expression of proangiogenic factors and MVD according to the histopathological type of ovarian cancer

	Serous ovarian cancer (*n* = 22)	Mucinous ovarian cancer (*n* = 8)	Endometrioid ovarian cancer (*n* = 7)	Clear cell ovarian cancer (*n* = 4)	Undifferentiated carcinoma (*n* = 17)	*P* value
VEGF Median pg/mg protein (range, min–max)	233.27 (0–3,000.0)	512.8 (27.25–2,240.4)	431.97 (166.20–2,298.3)	1,070.8 (325.57–1,433.1)	718.95 (10.14–3,000.0)	0.152
EMMPRIN Median ng/mg protein (range, min–max)	26.46 (10.65–88.22)	25.81 (8.48–106.50)	31.11 (13.35–60.43)	34.96 (27.19–69.75)	34.77 (10.86–90.28)	0.874
bFGF Median ng/mg protein (range, min–max)	1.03 (0.22–9.46)	1.54 (0.17–3.60)	0.81 (0.27–13.32)	2.03 (0.19–5.93)	0.94 (0.35–4.03)	0.833
CD105-MVD Median vessels/mm^2^ (range, min–max)	25 (0–38)	19 (0–51)	41 (13–51)	25 (0–44)	25 (0–57)	0.573
CD31-MVD Median vessels/mm^2^ (range, min–max)	73 (32–241)	70 (19–95)	101 (51–203)	60 (44–82)	63 (44–146)	0.541
CD34-MVD Median vessels/mm^2^ (range, min–max)	57 (6–165)	57 (44–108)	92 (57–146)	57 (38–70)	57 (6–190)	0.585

CD105-MVD, CD31-MVD, and CD34-MVD refer to MVD assessed with antibodies against CD105, CD31, and CD34, respectively

## Discussion

The results of our study indicate that EMMPRIN may contribute to the development of new blood vessels in ovarian cancer. Endoglin (CD105) is a well-established marker of active angiogenesis, as CD105 is expressed exclusively on newly formed vessels (Schliemann and Neri 2007; Dallas et al. 2008). By contrast, CD31 and CD34 are pan-endothelial markers that are found on endothelial cells of both new and mature vessels (Akagi et al. 2002). In the present study, we have shown a direct correlation between the expression of EMMPRIN and VEGF and MVD as assessed by antibodies against CD105. Similarly, we have revealed a direct correlation between the expression of VEGF and EMMPRIN. This result is supported by the previous findings in other neoplasms in which EMMPRIN was shown to stimulate the secretion of VEGF (Tang et al. 2005; Bougatef et al. 2009). However, in a logistic regression model, none of the analyzed proangiogenic factors independently indicated increased CD105-MVD. This may suggest that both molecules are important during the formation of new blood vessels in ovarian cancer.

Although the role of VEGF in ovarian cancer angiogenesis is well-established, there is limited data about the role of EMMPRIN (Yu et al. 2013). Millimaggi et al. (Millimaggi et al. 2007) demonstrated that microvesicles-containing EMMPRIN shed by ovarian cancer cell lines enhanced the proangiogenic activities of human umbilical vein endothelial cells (HUVECs). The stimulation of HUVECs by CD147-positive microvesicles increased invasiveness, the proliferation rate, MMP synthesis, and the formation of capillary-like structures. By contrast, microvesicles of low EMMPRIN concentration had a diminished ability to induce the proangiogenic phenotype of HUVECs (Millimaggi et al. 2007). In another study, Millimaggi et al. (Millimaggi et al. 2009) revealed that EMMPRIN expression in ovarian cancer cell lines is essential for vasculogenic mimicry (VM). VM is an alternative mechanism of angiogenesis in which tumor cells form tubes that act like microvessels. These channels are non-endothelial and are thus not relevant to our study, which was based on MVD evaluation (Millimaggi et al. 2009). VM is frequently associated with cancer aggressiveness (Sood et al. 2002). Indeed, in our study, we observed that EMMPRIN expression is higher in ovarian cancers diagnosed at an advanced stage and in grade 2/3 ovarian cancer when compared to grade 1 cancers. Similarly, Ueda et al. (Ueda et al. 2012) and Davidson et al. (2003) indicated that EMMPRIN expression was correlated with the poor prognosis of ovarian cancer patients. Thus, these results may support the relationship between EMMPRIN, VM, and the aggressiveness of ovarian cancer.

Various preclinical studies have demonstrated the proangiogenic properties of basic fibroblast growth factor. This growth factor can directly stimulate the proliferation and migration of endothelial cells, facilitate tube formation, sensitize ECs to other angiogenic factors, and stimulate the secretion of extracellular matrix remodeling proteases (Presta et al. 2005; Nissen et al. 2007). Giavazzi et al. (2003) suggest that bFGF and VEGF work synergistically to elicit angiogenesis. Interestingly, Alessi et al. (2009) demonstrated that targeting bFGF may overcome anti-VEGF resistance; thus, anti-bFGF therapy is undergoing clinical trials as an antiangiogenic therapy for ovarian cancer (Burger 2011). However, this is somewhat in contrast to our results, because we did not identify relationship between the expression of bFGF and the active angiogenesis. Therefore, we speculate that the timing of proangiogenic factors differs in the course of angiogenesis because VEGF appears earlier than bFGF (Lieu et al. 2011). Thus, increased EMPPRIN and VEGF expressions in malignant tumors associated with the upregulation of CD105 suggest the early phase and/or very active stimulation of angiogenesis in the studied malignant tumors.

Our study is the first to demonstrate an inverse correlation between the expression of EMMPRIN and bFGF in ovarian cancer tissue. Similar findings were obtained by Liu et al. (2011) in a study of head and neck cancer. The authors demonstrated that fibroblast growth factor receptor 2 (FGFR2) gene expression was inversely correlated with EMMPRIN expression. Moreover, Liu et al. (2011) showed that EMMPRIN-silenced tumors had more abundant stroma compared to controls. These results suggest that there could be an antagonistic interaction between EMMPRIN and bFGF. Evidence of the role of cancer-associated fibroblasts (CAFs) in the development and progression of cancer is increasing (Xing et al. 2010), and CAFs also play a substantial role in ovarian cancer (Schauer et al. 2011). Liu et al. (2011) suggest that tumor growth is fibroblast-dependent when EMMPRIN expression is low, while elevated EMMPRIN expression promotes fibroblast-independent tumor growth. Obermair et al. (1998) demonstrated that elevated tumor bFGF expression is correlated with favorable prognosis and that these tumors have greater stromal content. This is in agreement with the results of our study, because we have found higher bFGF expression in low-grade ovarian cancers, which tend to have better prognosis (Malpica et al. 2004). Additionally, we observed lower bFGF expression in ovarian cancer compared to benign ovarian tumors and normal ovaries. By contrast, some recent studies have shown that bFGF expression may be responsible for resistance to paclitaxel; thus, the exact role of bFGF as a prognostic factor in ovarian cancer is unclear (Gan et al. 2006). There appears to be close interactions between EMMPRIN and bFGF in the progression of ovarian cancer. However, exact prognostic significance of EMMRPIN/bFGF expression ratio should be verified according to survival analysis, and this is the main weakness of the presented study. We speculate that both EMMPRIN and bFGF may be key messengers between cancer cells and fibroblasts of the tumor stroma, but these interactions are likely very complex.

To conclude, the overexpression of EMMPRIN and VEGF in ovarian cancer creates a milieu of proangiogenic factors that may play a role in very early and/or reactivated angiogenesis. The upregulation of proangiogenic stimulants correlates with increased CD105-MVD; however, bFGF remains silenced. Additionally, high EMMPRIN/bFGF expression ratio is a new molecular profile of aggressive of EOC.
